# Genetic Deletion of Trace-Amine Associated Receptor 9 (TAAR9) in Rats Leads to Decreased Blood Cholesterol Levels

**DOI:** 10.3390/ijms22062942

**Published:** 2021-03-14

**Authors:** Ramilya Z. Murtazina, Ilya S. Zhukov, Olga M. Korenkova, Elena A. Popova, Savelii R. Kuvarzin, Evgeniya V. Efimova, Larisa G. Kubarskaya, Ekaterina G. Batotsyrenova, Ekaterina A. Zolotoverkhaya, Anastasia N. Vaganova, Sergey A. Apryatin, Natalia V. Alenina, Raul R. Gainetdinov

**Affiliations:** 1 Institute of Translational Biomedicine, Saint Petersburg State University, Universitetskaya nab., 7-9, 199034 Saint Petersburg, Russia; ramilya.murtazina@gmail.com (R.Z.M.); ilya.zhukov@skolkovotech.ru (I.S.Z.); lathyrus.pratense@gmail.com (O.M.K.); saveliy51@yandex.ru (S.R.K.); e.v.efimova@mail.ru (E.V.E.); van.inprogress@gmail.com (A.N.V.); apryatin@mail.ru (S.A.A.); alenina@mdc-berlin.de (N.V.A.); 2 Institute of Experimental Medicine, Acad. Pavlov str. 12, 197376 Saint Petersburg, Russia; 3 Max-Delbrück-Center for Molecular Medicine in the Helmholtz Association (MDC), Robert-Roessle-str. 10, 13125 Berlin, Germany; l.popova@mdc-berlin.de; 4 Institute of Toxicology of Federal Medical-Biological Agency, Bekhtereva str. 1, 192019 Saint Petersburg, Russia; larkub@yandex.ru (L.G.K.); bkaterina2009@yandex.ru (E.G.B.); orzheskovskaya@mail.ru (E.A.Z.); 5 Saint Petersburg State University Hospital, Saint Petersburg State University, Universitetskaya nab., 7-9, 199034 Saint Petersburg, Russia

**Keywords:** trace amines, trace amine-associated receptor, TAAR, TAAR9, low-density lipoprotein, cholesterol, animal knockout model, GPCR

## Abstract

In the last two decades, interest has grown significantly in the investigation of the role of trace amines and their receptors in mammalian physiology and pathology. Trace amine-associated receptor 9 (TAAR9) is one of the least studied members of this receptor family with unidentified endogenous ligands and an unknown role in the central nervous system and periphery. In this study, we generated two new TAAR9 knockout (TAAR9-KO) rat strains by CRISPR-Cas9 technology as in vivo models to evaluate the role of TAAR9 in mammalian physiology. In these mutant rats, we performed a comparative analysis of a number of hematological and biochemical parameters in the blood. Particularly, we carried out a complete blood count, erythrocyte osmotic fragility test, and screening of a panel of basic biochemical parameters. No significant alterations in any of the hematological and most biochemical parameters were found between mutant and WT rats. However, biochemical studies revealed a significant decrease in total and low-density lipoprotein cholesterol levels in the blood of both strains of TAAR9-KO rats. Such role of TAAR9 in cholesterol regulation not only brings a new understanding of mechanisms and biological pathways of lipid exchange but also provides a new potential drug target for disorders involving cholesterol-related pathology, such as atherosclerosis.

## 1. Introduction

Trace amine-associated receptors (TAARs) are a family of monoamine-related G-protein coupled receptors (GPCRs) discovered in 2001 by two independent groups of researchers [1,2]. They are represented by nine genes of members of this family in mammals but three of them are pseudogenes. Structurally, trace amines are closely related to well-known monoamine neurotransmitters such as dopamine, serotonin, and norepinephrine but their concentration in tissues is 100 times lower than that of these classic monoamine neurotransmitters. To date, TAAR1 is the most investigated member of the family with well-established expression in the brain and certain tissues in the periphery and is known to contribute to various CNS effects [3,4]. In 2019, the first drug based on TAAR1 agonism successfully passed Phase II of clinical trials for the treatment of schizophrenia [5].

Other TAARs (TAAR2, TAAR5, TAAR6, TAAR8, and TAAR9) were initially identified in the sensory neurons of the olfactory epithelium with projections to the olfactory bulb and until recently were believed to function exclusively as olfactory receptors sensing innate odors [4,6,7]. However, recent studies indicate that “olfactory” TAARs may also play important roles in higher CNS function by being expressed in limbic emotional brain areas receiving input from the olfactory bulb, as well as in periventricular areas and neurogenic zones [8,9]. As with other olfactory TAARs, TAAR9 is coupled to G_olf_-mediated cAMP accumulation [10]. Mouse TAAR9 is known to be activated by urine exposure of numerous mammalian species, including mouse, rat, human, and carnivores [10]. TAAR9 can be activated by tertiary amines (N-methylpiperidine and N, N-dimethylcyclohexylamine) but no cognate potent ligand has been identified so far [11]. Beyond olfactory epithelium, TAAR9 expression has been found in the human stomach [12], pituitary gland, and skeletal muscle [13]. TAAR9 mRNA has also been reported in the duodenal mucosal cells in the mouse gastrointestinal tract [14] and rat spinal cord [15]. As with most of the other TAARs, TAAR9 mRNA has been found in the spleen [16] and the full range of human leukocytes [17,18], indicating its potential role in hematological and immune processes.

Mouse and rat knockout models have become invaluable tools for studying the role of certain genes in mammalian physiology and pathology. While currently there are no reports on TAAR9 knockout animals, it is known that a mouse line with a cluster deletion (TAAR2-9) lacking all TAARs expressed in the olfactory epithelium showed no differences in comparison to WT with regard to fertility, litter size, distribution of genotype, and sex-ratio of pups, but demonstrated significant alterations in sexual behaviors related to the recognition of breeding partners and mate choice [19]. In this study, we generated two strains of TAAR9–KO rats using CRISPR/Cas9-mediated mutagenesis and focused on the analysis of hematological and basic biochemical parameters in the blood of TAAR9-KO rats by applying the protocol that we developed recently for TAAR1 knockout mice [20].

## 2. Results

### 2.1. Generation of TAAR9-KO Rats

**Figure 1:**
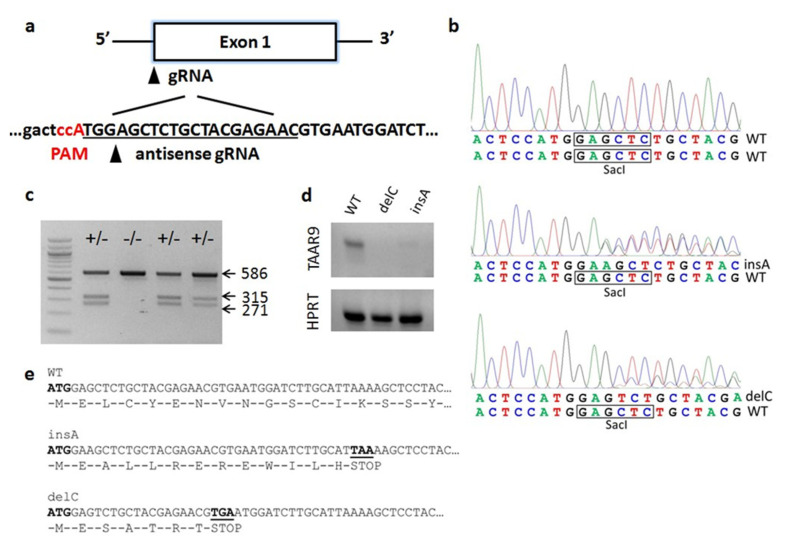
Generation of TAAR9-KO rats. (**a**) Diagram of the TAAR9 exon 1 sgRNA. The sgRNA sequence is underlined. Protospacer adjacent motif (PAM) sequence is indicated in red; the arrow indicates the Cas9 cutting site; the sequence in capital letters indicates the TAAR9 open reading frame. (**b**) Chromatograms of the TAAR9 genomic sequence from WT (upper panel), heterozygous insA (middle panel), and heterozygous delC (lower panel) animals. The black frame indicates the *SacI* restriction site on the WT allele. The *SacI* site was destroyed by both A insertion and C deletion. (**c**) Genotyping of TAAR9-KO rats generated by CRISPR-Cas9 technology by *SacI* digestion of PCR fragments. Undigested 586 bp band corresponds to the KO allele; the appearance of two bands, 315 bp and 271 bp, corresponds to a WT allele. The presence of all three bands corresponds to a heterozygous animal. (**d**) Reverse transcription-polymerase chain reaction (RT-PCR) with TAAR9- and HPRT (housekeeping gene)-specific primers using RNA isolated from the main olfactory epithelium (MOE) of WT, KO^delC^, and KO^insA^ rats. These gel images are cropped, and full-length images are presented in Supplementary Figure S1. (**e**). N-terminal TAAR9 protein sequences of the WT, KO^insA^, and KO^delC^ animals. Both A insertion and C deletion result in a premature stop codon and synthesis of short, non-functional peptides.

To generate knockout rats, we targeted rat TAAR9 gene by using CRISPR-Cas9 genome editing. A single guide RNA (sgRNA) and protospacer adjacent motif (PAM) was designed to target the coding strand of the rat TAAR9 gene with a predicted cut site 5 bp downstream of the translation initiation codon (Figure 1a). A total of 31 embryos microinjected with sgRNA and Cas9 mRNA were implanted to foster mothers that resulted in the birth of 17 founder offspring, which were analyzed for edits. Two founders carried mutations in the targeted region, deletion of cytosine, and insertion of adenine (Figure 1b), and gave rise to two TAAR9-KO rat lines, TAAR9-KO^delC^ and TAAR9-KO^insA^, respectively, and loss of the *SacI* restriction site (Figure 1b), that was used to distinguish WT and KO alleles for the genotyping of the animals (Figure 1c). The selected founders were backcrossed to WT rats to generate the heterozygous (HET) F1 and F2 generations. While backcrossing of the strains is being continued to exclude potential “off-target” effects of CRISPR-Cas9-based genetic manipulations [21], we expanded a colony of F2 generation to perform these studies. By observing similar alterations in two independently developed strains of knockout of the same gene, we are excluding the likelihood of contribution of “off-target” unwanted mutation to the effects observed.

### 2.2. Validation of TAAR9-KO Rats

WT, HET, and KO animals for the TAAR9 of both strains were born at close to Mendelian ratios, and homozygous KO animals were viable and reached adulthood without any overt phenotypes. Next, we performed validation of the TAAR9-KO animals at the mRNA level. mRNA was isolated from an olfactory epithelium (OE), the site with the highest TAAR9 expression, of WT and both KO^insA^ and KO^delC^ TAAR9-KO strains, reverse-transcribed into cDNA, and quantified by qPCR. Although housekeeping transcripts (HPRT) were identified in all probes analyzed, TAAR9 mRNA was expressed only in WT animals, but was undetectable in mutant rats by qPCR. Additionally, a drastic reduction in TAAR9 expression was visible when PCR products were run on the agarose gel (Figure 1d). Both mutations lead to a reading frame shift resulting in a premature stop codon (Figure 1e). The insertion of a premature termination codon in exon 1 in both strains may result in a loss of mRNA expression likely due to the nonsense-mediated decay of mRNA [22].

### 2.3. Hematological Analysis of TAAR9-KO Rats

**Figure 2:**
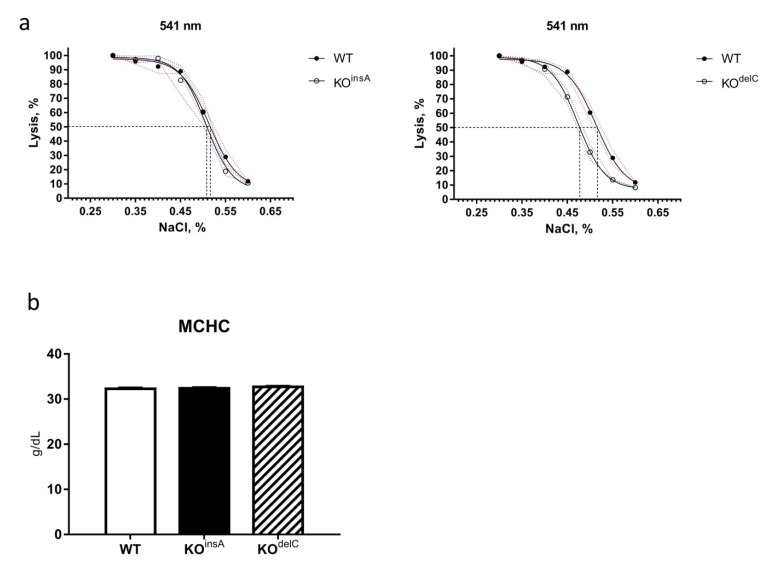
Comparative osmotic fragility test (OFT) and mean corpuscular hemoglobin concentration (MCHC) analysis of WT and TAAR9-KO rats. (**a**) Differences in the hemolysis of red blood cells (RBC) at different concentrations of NaCl are detectable only on KO^delC^ strain. Evaluation results of 50% lysis concentration point: WT= 0.518, KO^insA^ = 0.509, KO^delC^ = 0.479; *n* = 5 per group (**b**) No alterations in mean corpuscular hemoglobin concentration (MCHC) between TAAR-KO and WT animals; *n* = 7 per group. Data are mean ± SEM.

To evaluate if the lack of TAAR9 affects physiological functions we first performed the hematological analysis in these rats. Osmotic fragility test (OFT) is one of the standard clinical tests for diagnosis of blood pathologies such as hereditary spherocytosis, autoimmune hemolytic anemia, and other erythrocyte abnormalities [23]. Hemolysis of RBC is a process of releasing hemoglobin into the plasma as a result of a ruptured erythrocyte membrane. Osmotic fragility is affected by various factors, including membrane composition and integrity, as well as the cell sizes or surface-area-to-volume ratios [24]. To evaluate the potential role of TAAR9 in erythrocyte pathologies, we tested both strains of TAAR9-KO rats in the OFT test. Whereas TAAR9-KO^delC^ rats showed a reduced percentage of hemolysis in comparison to WT, this phenotype was not confirmed in the TAAR9-KO^insA^ strain (Figure 2a). Furthermore, no differences between mean corpuscular hemoglobin concentration (MCHC) levels were observed between genotypes (Figure 2b). From these results, we conclude that the lack of the TAAR9 gene is not causing prominent spherocytosis pathology on the stage of differentiated erythrocytes.

**Figure 3:**
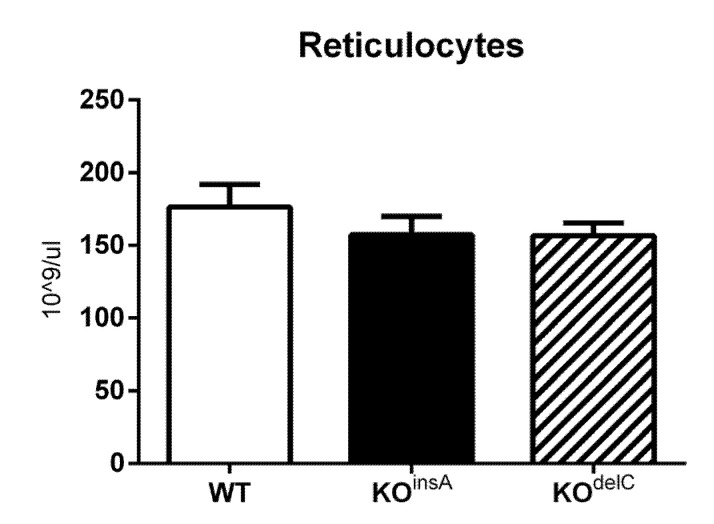
Reticulocyte levels of TAAR9-KO and WT rats. There are minor trends detected in reticulocyte cell concentration in TAAR9-KO strains in comparison to WT rats, *n* = 7 per group. Data are mean ± SEM.

We further investigated if TAAR9 deletion affects erythropoiesis processes. In our study, we used an automatic analyzer to measure reticulocytes, the progenitors of red blood cells that detected them with a special RETIC solution for Advia2120i (Siemens, München, Germany). As presented in Figure 3, there were no significant differences between the three groups but a minor trend for a decrease in the number of reticulocytes was noted in both TAAR9-KO rat strains.

**Figure 4:**
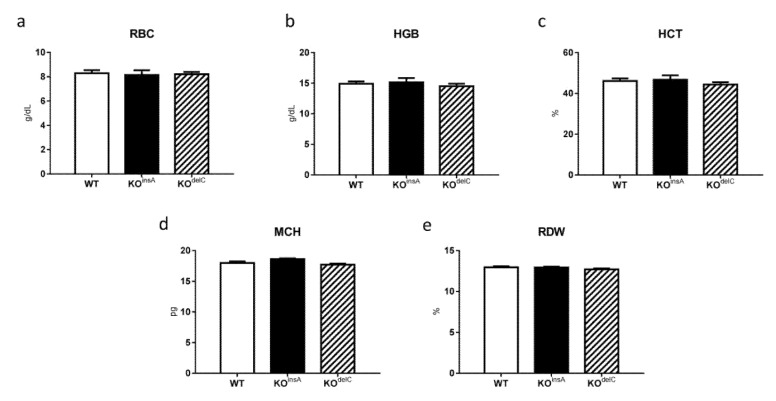
Hematology parameters of WT and TAAR9-KO rats. Minimal changes between genotypes were detected in (**a**) red blood cells (RBC), (**b**) hemoglobin (HGB), (**c**) hematocrit (HCT), (**d**) mean corpuscular hemoglobin (MCH), (**e**) red blood cell distribution width (RDW). Data are mean ± SEM, *n* = 7 per group.

Other RBC measurements, including red blood cell count (RBC), hemoglobin (HGB), hematocrit (HCT), mean corpuscular hemoglobin (MCH), red blood cell distribution width (RDW), showed minimal changes between genotypes (Figure 4).

**Figure 5:**
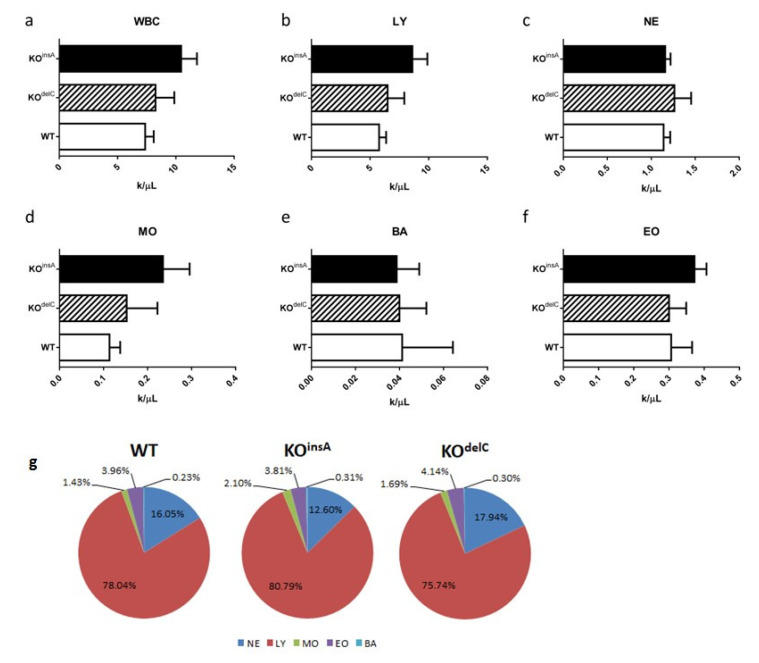
Rat white blood cells (WBC) parameters and WBC differential of WT and TAAR9-KO rats. (**a**–**f**) Comparative analyzes revealed minimal alterations in immune parameters: (**a**) white blood cells (WBC) in total, (**b**) lymphocytes (LY), (**c**) neutrophils (NE), (**d**) monocytes (MO), (**e**) basophils (BA), (**f**) eosinophils (EO). Data are mean ± SEM; (**g**) diagrams present no changes in both TAAR9-KO groups in comparison to WT rats. NE: neutrophils, LY: lymphocytes, MO: monocytes, EO: eosinophils; BA: basophils. Data are mean (%), *n* = 7 per group.

WBC (Figure 5a–f) and WBC differential (Figure 5g) of TAAR9-KO and WT rats were analyzed [20]. Results of WBC analysis are presented as concentration (Figure 5a–f) and leukocyte formula view (Figure 5g). Generally, there were only minimal alterations between both TAAR9-KO groups and WT. Although there was a minor trend to a higher number of WBC, lymphocytes, monocytes, and basophils in both TAAR9-KO strains, these changes did not reach statistical significance. Full hematological results are presented in the supplementary materials (Supplementary Table S1).

### 2.4. Biochemical Analysis of TAAR9-KO Rats

**Figure 6:**
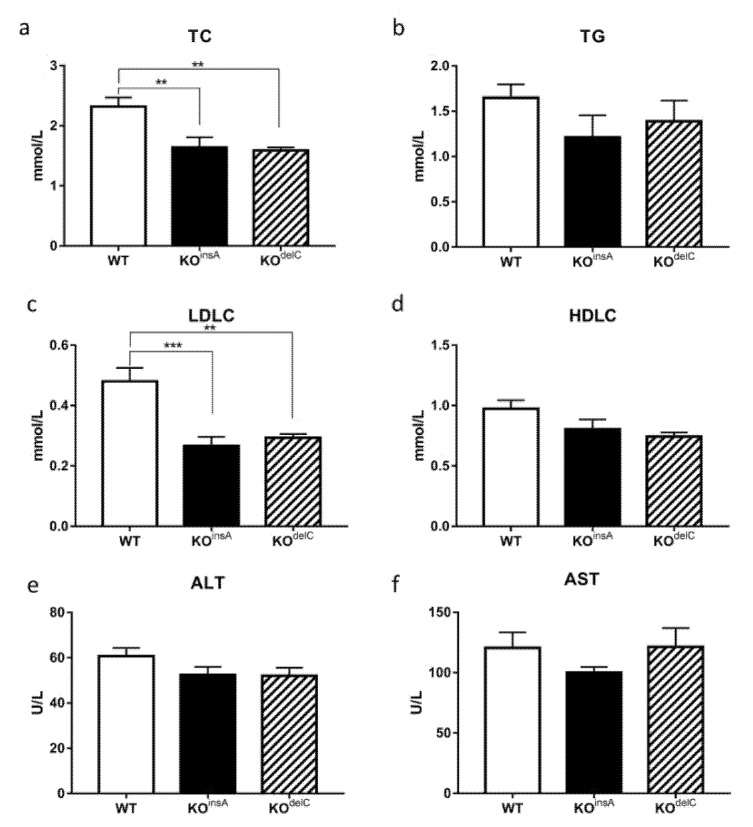
Comparative analysis of biochemical parameters in the blood of TAAR9-KO and WT rats. (**a**) Total cholesterol (TC), (**b**) triglycerides (TG), (**c**) low-density lipoprotein cholesterol (LDLC), (**d**) high-density lipoprotein cholesterol (HDLC), (**e**) alanine aminotransferase (ALT), (**f**) aspartate aminotransferase (AST). The biochemical screening revealed changes in several parameters of lipid regulation. Data are mean ± SEM, ** *p* < 0.01, *** *p* < 0.001 vs. WT, ANOVA with Tukey’s post-hoc test, *n* = 7 per group.

**Figure 7:**
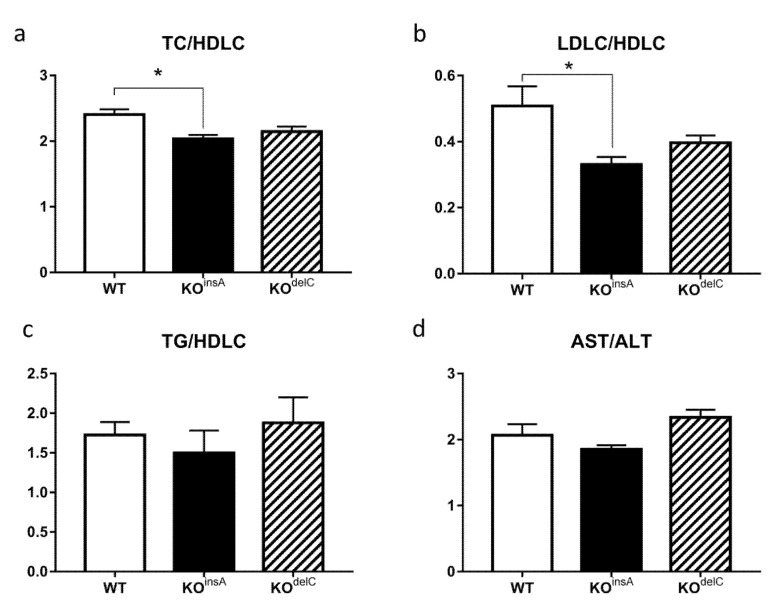
Comparative analysis of biochemical parameters in the blood of TAAR9-KO and WT rats. (**a**) Total cholesterol/high-density lipoprotein ratio (TC/HDLC), (**b**) ratio of low-density lipoprotein cholesterol and high-density lipoprotein cholesterol (LDLC/HDLC), (**c**) ratio of triglyceride/high-density lipoprotein cholesterol (TG/HDLC), (**d**) de Ritis ratio (AST/ALT). Biochemical analysis revealed a decrease in TC-to-HDLC ratio and LDL to HDL cholesterol, the main predictive factor of atherosclerosis in human [25], in TAAR9-KO rats, but statistical significance was observed only in TAAR9-KO^insA^ strain. Data are mean ± SEM, * *p* < 0.05 vs. WT, ANOVA with Tukey’s post-hoc test, *n* = 7 per group.

Comparative blood biochemical analysis of TAAR9-KO and WT rats revealed changes in metabolic and lipid regulation. Total cholesterol (TC) was significantly decreased in both TAAR9-KO lines (KO^insA^ 1.64 ± 0.13 mmol/L; KO^delC^ 1.59 ± 0.45 mmol/L vs. WT 2.32 ± 0.40 mmol/L, *p* < 0.01, Figure 6a). Furthermore, a trend to lower blood triglycerides (TG) levels were observed in both TAAR9-KO models, although these differences did not reach statistical significance (Figure 6b). We further investigated if low-density lipoprotein cholesterol (LDLC) or high-density lipoprotein cholesterol (HDLC) have contributed to the drop in cholesterol levels. Whereas HDLC levels were not affected by lack of TAAR9 (Figure 6d), LDLC levels mirrored the differences observed in TC, being decreased by half in TAAR9-KO animals in comparison to WT rats (KO^insA^ 0.27 ± 0.08 mmol/L; KO^delC^ 0.26 ± 0.09 mmol/L vs. WT 0.48 ± 0.12 mmol/L, *p* < 0.01, Figure 6c). Importantly, TC-to-HDLC ratio, the main predictive factor of atherosclerosis in human [25], was decreased in TAAR9-KO rats, but significance was observed only in TAAR9-KO^insA^ strain (Figure 7a).

Additionally, we calculated LDLC/HDLC and TG/HDLC ratio. LDL/HDL cholesterol ratio was decreased in TAAR9-KO, but only TAAR9-KO^insA^ reached statistical significance (Figure 7b). The ratio of triglycerides to HDL-cholesterol (TG/HDLC) was not altered in TAAR9 knockout rats (Figure 7c).

Investigation of ALT, a clinical biomarker for liver health [26], revealed a slight decrease in TAAR9-KO rats (Figure 6e). However, no significant changes were observed for aspartate aminotransferase AST (Figure 6f) and AST/ALT ratio (de Ritis ratio) (Figure 7d), indicating a lack of major pathological processes in the liver or cardiovascular system [27].

Furthermore, no differences in other biochemical parameters such as PL and LDH, as well as in total protein, phosphor, glucose, creatinine, total bilirubin, albumin, and urea (Supplementary Table S2) were observed in TAAR9-KO rats in comparison to WT animals.

## 3. Discussion

In this study, we report the generation of two TAAR9-KO rat strains, independently produced via CRISPR/Cas9-mediated gene editing in rat zygotes. Both generated strains carried a single-nucleotide insertion/deletion mutation leading to the frameshift of the open reading frame of TAAR9 and drastically decreased TAAR9 mRNA levels. To understand the role of TAAR9 in mammalian physiology, in this study, we focused on the evaluation of hematological and biochemical blood parameters in TAAR9-KO^delC^ and TAAR9-KO^insA^ strains of rats lacking TAAR9. We elected to investigate two independently developed strains of knockout of the same gene to exclude the contribution of potential “off-target” unwanted mutations, that might be created by CRISPR/Cas9 genome editing [21], to the effects observed.

A number of hematological parameters evaluated were not significantly altered in rats lacking TAAR9. All erythrocyte parameters tested (MCHC, RBC, HCT, HGB, etc.) had only minor deviations in mutants in comparison to WT controls; OFT also did not reveal any hidden spherocytosis pathologies. No changes in WBC, WBC differential levels, and other routine hematological parameters were found in mutant rats. Thus, no evidence for the role of TAAR9 in the regulation of any hematological parameter was found suggesting that future TAAR9-based therapies could have minimal hematological side effects.

At the same time, we found significant physiological changes in lipid homeostasis in animals lacking TAAR9. Firstly, TC levels were significantly decreased in both strains of TAAR9-KO rats. Furthermore, we clarified that this drop in TC levels was mostly caused by the decrease in LDL fraction, increased levels of which are known to be critically involved in the pathogenesis of atherosclerosis [28]. The dysregulation of cholesterol homeostasis contributes to the pathogenesis of several alimentary-dependent diseases [28]. The concentration of cholesterol in the blood is an important metabolic biomarker not only for atherosclerosis and its reduction can be found in certain disorders, such as hyperthyroidism, during the damage of the central nervous and immune systems, and some other multiple organ dysfunctions [29,30,31]. It is also known that endogenous and exogenous cholesterol is metabolically involved in cell membrane production [32]. While regulation of lipid metabolism during erythropoiesis is poorly understood, the decreased cholesterol level may potentially affect reticulocyte maturation processes [32]. However, in our study, total cholesterol deficiency did not cause major disruptions in the development and function of major groups of blood cells.

The well-known total/high-density lipoprotein cholesterol ratio (TC/HDLC), described also as the atherogenic or Castelli index, as well as the LDL/HDL cholesterol ratio are two commonly used indicators of cardiovascular risk [25]. These ratios have a predictive value greater than just measures of individual parameters. It is well established that individuals with a high total/HDL cholesterol or LDL/HDL cholesterol ratio have significantly higher cardiovascular risk. This is explained by an imbalance between the cholesterol represented by atherogenic and protective lipoproteins due to an increase in the atherogenic component or a decrease in the protective component, or both [25]. Both TC/HDLC and LDL/HDL cholesterol were decreased in TAAR9-KO, but only in TAAR9-KO^insA^ this difference reached statistical significance. The ratio of triglycerides to HDL-cholesterol (TG/HDLC), an indicator of coronary disease [33], was not altered in TAAR9 knockout rats. Similarly, measurements of AST, ALT, and AST/ALT ratio (de Ritis ratio) indicated a lack of major pathological processes in the liver or cardiovascular system. Since ALT is critically involved in amino acid metabolism and gluconeogenesis in the liver, ALT measurement is used as a valid indicator of hepatotoxicity [26]. While ALT is primarily found in the liver, with considerably less expression detected in the skeletal muscle and heart tissue, AST is found in the heart, brain, skeletal muscle, and, to a lesser degree, in the liver [34]. Damage to hepatocytes results in the release of their contents including ALT into the extracellular space that ultimately causes an increase in the serum levels of ALT. At the same time, since AST is mostly reflective of the damage to myocytes, the ratio of serum AST to ALT is commonly used to differentiate liver damage from the damage of other tissues [26]. Lack of significant changes in AST, ALT, and AST/ALT ratio as well as other biochemical parameters in both mutants indicate low likelihood of these pathological processes in rats lacking TAAR9.

The decrease in blood cholesterol levels (TC and LDLC) could be explained by several reasons. It may occur due to decreased endogenous cholesterol biosynthesis, transport from the liver to organs, or alterations in metabolism [28]. Furthermore, since TAAR9 is known to be expressed in the mouse gastrointestinal tract [14], it potentially can be involved in the regulation of exogenous lipid absorption, including cholesterol and triglycerides in the small intestine [28], although we could not detect expression of TAAR9 mRNA by RT-PCR in rat duodenum and caecum (data not shown). Additionally, we performed a meta-analysis of published RNAseq datasets (see Supplementary Analysis 1) and found low or below-cutoff TAAR9 expression in human pancreatic islets and adipose tissue, as well as in mouse enterocytes of large intestine. Further detailed studies are necessary to evaluate the molecular mechanisms involved. Nevertheless, these data suggest an unexpected role of TAAR9 in the regulation of cholesterol homeostasis that may have important clinical and pharmacological implications. Thus, more attention should be paid to the evaluation of the role of other TAARs in lipid and cholesterol metabolism, in general. Intriguingly, trimethylamine (TMA), a by-product of bacterial degradation of dietary choline, betaine, L-carnitine, and phosphatidylcholine in the gut, potently activates another member of TAAR family, TAAR5 [35,36]. TMA metabolite trimethylamine *N*-oxide (TMAO) has been shown to play a role in the pathology of several human disorders, including cardiovascular diseases. It was also implicated in the reverse cholesterol transport, and glucose and lipid homeostasis, in general [37]. Increased TMAO levels are found in individuals with obesity and this measure is used to predict the risk of atherosclerosis. While the molecular mechanism by which TMAO contributes to these pathologies is currently unknown, it may involve alterations in the cholesterol homeostasis [37]. Whether altered TAAR5 signaling contributes to these conditions is unknown, however, it has been shown that trimethylamine-N-oxide can be converted back to TMA by gut microbiota [38]. Furthermore, 3-iodothyronamine (T1AM), an agonist of another member of the TAAR family, TAAR1, has been investigated as a potential anti-obesity drug affecting fatty acid catabolism and pyruvate toward gluconeogenesis in the liver and adipose tissue. T1AM is shown to upregulate several genes involved in lipoprotein functions in the adipose tissue and genes related to lipid metabolism and cholesterol uptake in the liver [39].

Finally, the association between high LDLC levels and atherosclerosis has been well established [40]. Major clinical manifestations of atherosclerosis include ischemic heart disease, the leading cause of premature adult mortality worldwide [41]. LDLC deposition causes the formation of well-defined lesions in the arterial intima that, as a consequence, can precipitate clinical events such as heart attack and stroke [25,28]. Thus, finding new therapeutical targets to reduce LDLC levels may represent the immediate practical interest and result in the development of a new class of pharmacological drugs for atherosclerosis treatment.

In conclusion, our observations suggest that future medications based on TAAR9 antagonism may have potential in the treatments of disorders related to elevated cholesterol levels such as atherosclerosis. At the same time, relatively minor alterations in most hematological and biochemical parameters in rats lacking TAAR9 may indicate a good safety profile of such future medications.

## 4. Materials and Methods

### 4.1. Generation of TAAR9-Knockout Rats

TAAR9-KO rats were produced using CRISPR/Cas9-based technology on the Sprague Dawley outbred genetic background. Single guide RNA (sgRNA) was designed to target the translation initiation site at the TAAR9 single exon according to the Zhang Lab protocols [42]. To prepare the TAAR9-sgRNA expression plasmid, the oligo pairs 330TAAR95 (5′-CACCGTTCTCGTAGCAGAGCTCCA-3′)/330TAAR93 (5′-AAACTGGAGCTCTGCTACGAGAAC-3′) encoding the 20-nt guide sequences (5′-GTTCTCGTAGCAGAGCTCCA-3′) were annealed and ligated into pX330 plasmid (Addgene plasmid ID: 42230) digested with *BbsI*. The sgRNA was amplified with primers T7sgRNATAAR9fw (5′-TTAATACGACTCACTATAGGTTCTCGTAGCAGAGCTCCA-3′) and sgRNA31 (5′-AAAAGCACCGACTCGGTGCC-3′) to add a T7 promoter sequence. sgRNA was transcribed in vitro by T7 RNA polymerase using Ambion MEGA shortscript T7 kit (AM1354). A mixture of sgRNA (25 ng/µL) and *Cas9* mRNA (IDT, 30 ng/µL) was microinjected into pronuclei of fertilized eggs from Sprague Dawley rats (Janvier, Le Genest-Saint-Isle, France). The injected zygotes were cultured to the 2-cell stage and transferred into pseudopregnant females according to established methods [43]. The resulting offspring were genotyped by polymerase chain reaction with primers flanking the gRNA target region (Taar95: 5′-CAGCAGGTAGAAAATGGGAG-3′ and Taar931 5′-AGTGGAGGATAGCGGTGATG-3′) and screened by Sanger sequencing.

### 4.2. Genotyping

Two mutant lines, with a single nucleotide insertion (TAAR9-KO^insA^) and single nucleotide deletion (TAAR9-KO^delC^), were used in the study. Since both mutations resulted in a loss of *SacI* restriction site, genotyping of animals was performed as a combination of PCR followed by *SacI* digestion. PCR was run on genomic DNA isolated from tail biopsies using primers flanking the mutation site (TAAR952: 5′-TGGCCTTTTGCAAGAAGTTT-3′ and TAAR932: 5′-GCAAAGCAGAAGGAGGTGTC-3′). PCR amplification was performed in a buffer containing 2 × BioMaster HS-Taq PCR-Color reaction mix (Biolabmix, Russia) with 1 μM of each primer, and 500 ng genomic DNA. After incubation at 95 °C for 3 min, 35 cycles of 20 s at 95 °C, 20 s at 63 °C, 40 s at 72 °C, and a final incubation of 72 °C at 5 min, the resulting 586 bp product was digested by *SacI* restriction enzyme (Thermo Scientific, Boston, MA, USA). The appearance of two bands at 315 and 271 bp corresponds to the WT; of three bands at 586, 315, and 271 bp to HET; and of single undigested 586 bp band to KO genotypes. The genotypes of all rats used in this study were confirmed twice, from samples collected at weaning and after the sacrifice.

### 4.3. Gene Expression Analysis by qPCR

RNA isolation from the olfactory epithelium (OE) was performed using TRIzol (TRIzol Reagent, Invitrogen, Darmstadt, Germany) according to the manufacturer’s instructions. The RNA pellet was resuspended in RNase-free water and kept at −80 °C until used. RNA concentration was quantified using spectrophotometry (NanoDrop, München, Germany). To eliminate any remaining genomic DNA gDNA Eraser DNA Removal Kit (Grisp, Spain) was used on RNA samples. Thus, 1 *μ*g of RNA was taken for the synthesis of cDNA using Reverse Transcription Kit (Evrogen, Moscow, Russia). In brief, reverse transcriptase and reaction mix containing 1 μM random primers were added to DNAse-treated RNA and exposed to the following protocol: annealing at 25 °C for 10 min, transcription at 42 °C for 90 min, and termination at 70 °C for 10 min. cDNA samples were stored at −20 °C. As a control for successful removal of genomic DNA, each sample was exposed to the same treatment except that the reverse transcriptase was not added (RT^−^ control). Gene expression was assessed using qPCR in Real-Time CFX96 (Bio-Rad, Hercules, CA, USA). Reactions were performed in triplicates using qPCRmix-HS SYBR (Evrogen, Russia) under the following condition: 95 °C for 5 min followed by 35 cycles of 95 °C for 20 s, 60 °C for 20 s, and 72 °C for 30 s. Primers were designed to detect TAAR9 (TAAR93_fw: 5′-AAGAGTAGCCAGACGAGAGAGG-3′, TAAR93_rev: 5′-TCATGTAGGCATCAATCACGGC-3′) and were tested for specific target amplification in the sample compared to the RT^−^ control using melting curve analysis (from 55 to 95 °C) and 3% agarose gel electrophoresis. Hypoxanthine-guanine phosphoribosyl transferase gene (primers: ratHPRT-RT F: 5′-CTC ATGGACTGATTATGGACAGGAC-3′, ratHPRT-RT R: 5′-GCAGGTCAGCAAAGAACTTATAGCC-3′), which is a housekeeping gene, was included as an internal control [9].

### 4.4. Animals

Rats were maintained on a 12:12 h light/dark cycle with a light on at 08:00 h, and allowed access to food and water ad libitum. All procedures were performed under the guidelines established by the European Community Council (Directive 2010/63/EU of 22 September 2010) and animal protocols were approved by responsible governmental authorities (Landesamt für Gesundheit und Soziales (LaGeSo), Berlin, Germany, and Ethics Committee of St. Petersburg State University, St. Petersburg, Russia). *Three months-old Sprague Dawley* TAAR9-KO rats (TAAR9-KO*^insA^* and TAAR9-KO^delC^ strains) and wild type (WT) littermates were used in the study.

### 4.5. Sample Collection and Storage

Whole blood (postprandial) for hematological parameters and osmotic fragility test (OFT): rats were shortly anesthetized with isoflurane and killed by decapitation. Trunk blood was placed into VACUETTE K3-EDTA tubes (Greiner Bio-One, Kremsmünster, Austria), and stored at +4 °C or room temperature before the experiment (samples were analyzed within 1–3 h after blood collection). After hematological measurement, red blood cells (RBC) of each sample were isolated for osmotic fragility test [20].

Serum for biochemical screening (postprandial): rats were shortly anesthetized with isoflurane and killed by decapitation, trunk blood was collected into VACUETTE blood collection tubes for serum (Greiner Bio-One, Austria), incubated in a vertical position for 15 min, and then kept at +4 °C until centrifugation. Samples with coagulated blood were centrifuged at 1500 rpm for 15 min at +4 °C. Serum was transferred into dry clean tubes and stored until analysis at −20 °C for no more than 5 days.

### 4.6. Measurement of Hematological Parameters

Hematological tests were performed using an automated hematology analyzer ADVIA 2120i (Siemens Healthcare Diagnostics, Eschborn, Germany) as described in [20]. This flow cytometry-based system uses light scatter, differential white blood cell (WBC) lysis, and myeloperoxidase and oxazine 750 staining to provide a complete blood cell (CBC), a WBC differential, and reticulocyte counts. A cyanide-free method is used to measure hemoglobin colorimetrically.

Hematological parameters such as CBC parameters and leukocyte subpopulations, erythrocyte count (RBC), hemoglobin, hematocrit (HCT), reticulocytes (Retic), mean corpuscular volume (MCV), mean corpuscular hemoglobin (MCH), mean corpuscular hemoglobin concentration (MCHC), red blood cell distribution width (RDW), platelets (PLT), white blood cells (WBC), neutrophils (NE), lymphocytes (LY), monocytes (MO), eosinophils (EO), and basophils (BA) were measured [20].

### 4.7. Osmotic Fragility Test (OFT)

Rat sample blood was taken in VACUETTE K3-EDTA tubes (Greiner Bio-One, Austria) and RBC mass was purified via centrifugation at 1500 rpm for 15 min at +4 °C. Then, 5 µL of the cell suspension was transferred at room temperature to tubes containing 2.5 mL each of graded concentrations of NaCl (0.25, 0.35, 0.45, 0.55, 0.65%). The tubes were gently mixed and after incubation for 10 min at room temperature, the non-lysed red cells were removed by centrifugation at 1500 rpm for 15 min at +4 °C.

The relative amount of hemoglobin released into the supernatant was determined spectrophotometrically (Beckmann Coulter DU 800, USA) at 541, 555, 577 nm, with a 0.85% NaCI sample serving as a blank and a 0.1% NaCl sample serving as the 100% lysis point [20].

### 4.8. Measurement of Biochemical Parameters

For biochemical screening of TAAR9 knockout rats, automatic analyzer Random Access A-25 (Biosystems S.A., Barcelona, Spain) was used, utilizing the spectrophotometer principle. Serum samples were stored at −20 °C before analysis. The following biochemical parameters were analyzed: total cholesterol (TC), low-density lipoprotein cholesterol (LDLC), high-density lipoprotein cholesterol (HDLC), total protein, phosphorus, glucose, creatinine, total bilirubin, alanine aminotransferase (ALT), albumin, aspartate aminotransferase (AST), lactate dehydrogenase (LDH), lipase (PL), urea, triglycerides (TG).

Significantly altered parameters were confirmed on an additional analyzer (Olympus AU400, Tokyo, Japan) with a different set of reagents to exclude methodological mistakes [44].

### 4.9. Interassay Repeatability

Before analysis, equipment was decontaminated, calibrated, and checked by internal quality control. Interassay repeatability was estimated by calculating the coefficient of variation (CV) of 10 consecutive measurements of internal quality control material in three different controls (low, normal, and high). CV (expressed in %) was calculated as standard deviation (SD)/mean × 100 [20].

### 4.10. Statistical Analysis

Values obtained are expressed as mean ± SEM and were further subjected to one-way analysis of variance (ANOVA) with post-hoc Tukey HSD test, using GraphPad Prism version 6.0 for Windows from GraphPad Software, San Diego, CA, USA (http://www.graphpad.com, accessed on 2 March 2021). Values of *p* < 0.05 were considered to be significant.

## Data Availability

All of the data is presented in the article and supplementary files. No additional data is reported.

## Supplementary Materials

The following are available online at https://www.mdpi.com/1422-0067/22/6/2942/s1, Figure S1: Full-length agarose gel: Reverse transcription-polymerase chain reaction (RT-PCR) with TAAR9 and HPRT (housekeeping gene), Table S1: Comparative hematological analysis of TAAR9-KO and WT rats, Table S2: Comparative blood biochemical analysis of TAAR9-KO and WT rats, Supplementary Meta-analysis 1: TAAR9 expression in organs involved in cholesterol metabolism regulation (RNAseq datasets meta-analysis).
