# Supplementary material for: Genetic Deletion of Trace-Amine Associated Receptor 9 (TAAR9) in Rats Leads to Decreased Blood Cholesterol Levels
Source: Int J Mol Sci. 2021 Mar 14;22(6):2942. doi: 10.3390/ijms22062942 (PMC7998418; doi:10.3390/ijms22062942)
Supplement: Supplementary file 1 [file ijms-22-02942-s001.pdf]

Article

Genetic deletion of trace-amine associated receptor 9 (TAAR9) in rats leads to decreased blood cholesterol levels

Ramilya Z. Murtazina [1+], Ilya S. Zhukov [1,2+], Olga M. Korenkova[1], Elena N. Popova [3], Savelii R. Kuvarzin [1], Evgeniya V. Efimova [1], Larisa G. Kubarskaya [4], Ekaterina G. Batotsyrenova [4], Ekaterina A. Zolotoverkhaya [4], Anastasia N. Vaganova[1], Sergey A. Apryatin [1,2], Natalia V. Alenina [1,3], and Raul R. Gainetdinov [1,5*]

[1] Institute of Translational Biomedicine, Saint Petersburg State University, Universitetskaya nab., 7-9, Saint Petersburg, 199034, Russia; dopamines@gmail.com

[2] Institute of Experimental Medicine, Acad. Pavlov str. 12, Saint Petersburg, 197376, Russia;

[3] Max-Delbrück-Center for Molecular Medicine in the Helmholtz Association (MDC), Berlin, Germany

[4] Institute of Toxicology of Federal Medical-Biological Agency, Bekhtereva str. 1, Saint Petersburg, 192019, Russia

[5] Saint Petersburg State University Hospital, Saint Petersburg State University, Universitetskaya nab., 7-9, Saint Petersburg, 199034, Russia

* Correspondence: gainetdinov.raul@gmail.com; Institute of Translational Biomedicine, Saint Petersburg State University, Universitetskaya nab., 7-9, Saint Petersburg, 199034, Russia

\+ these authors contributed equally to the study

SUPPLEMENTARY MATERIALS

Supplementary Figure 1. Full-length agarose gel: reverse transcription-polymerase chain reaction (RT-PCR) with TAAR9 and HPRT (housekeeping gene) specific primers using RNA isolated from the main olfactory epithelium (MOE) of WT, KO^{delC}, and KO^{insA} rats confirmed a corresponding decrease in TAAR9 mRNA expression in the main olfactory epithelium (MOE) of both KO strains. Negative control is K-. Red is a source of cropped image.

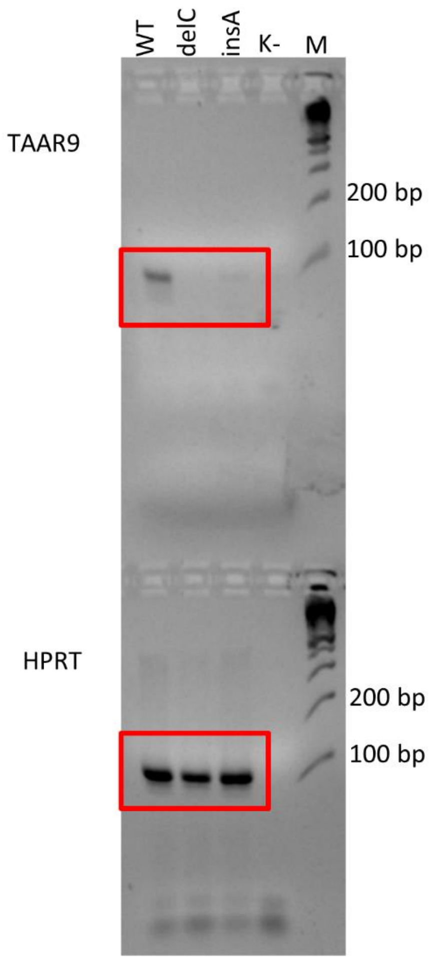

Supplementary Table 1. Comparative hematological analysis of TAAR9-KO and WT rats. Data are Mean ± SEM, n=7 per group.

	Parameters (units)	WT	TAAR9-KO^{insA}	TAAR9-KO^{delC}
Basic parameters	MCV(fL)	55,63 ± 0,72	57,50 ± 0,45	54,06 ± 0,39
	CHCM(g/dL)	32,44 ± 0,27	32,40 ± 0,18	33,03 ± 0,20
	CH(g/dL)	18,01 ± 0,27	18,61 ± 0,13	17,88 ± 0,19
	HDW(g/dL)	2,78 ± 0,39	2,98 ± 0,04	3,14 ± 0,03
	PLT(10*3/µl)	524,75 ± 80,36	445,13 ± 40,66	459,38 ± 56,83
	MPV(fL)	9,26 ± 0,49	9,49 ± 0,53	9,39 ± 0,32
WBC differential	LUC	0,26 ± 0,07	0,36 ± 0,08	0,20 ± 0,06
	LI	2,34 ± 0,02	2,35 ± 0,04	2,35 ± 0,04
	MPXI	30,53 ± 6,58	40,18 ± 5,64	20,48 ± 5,99
	WBCP(10*3/µl)	8,46 ± 0,85	11,48 ± 1,32	8,95 ± 1,57
Reticulocytes	Retic(%)	2,14 ± 0,09	2,07 ± 0,16	1,92 ± 0,13
	CHr(pg)	23,64 ± 0,35	24,25 ± 0,08	22,95 ± 0,26
	CHm(pg)	23,99 ± 2,04	26,75 ± 2,49	25,99 ± 2,76
Additional parameters	%Hyper	3,30 ± 0,54	2,30 ± 0,38	3,43 ± 0,32
	%Hypo	0,30 ± 0,04	0,23 ± 0,03	0,20 ± 0,03
	RBC Fragments(10*6 /µl)	0,05 ± 0,00	0,04 ± 0,00	0,04 ± 0,01
	RBC Ghosts(10*6/µl)	0,02 ± 0,00	0,02 ± 0,00	0,02 ± 0,00
	Neut X	21,11 ± 0,14	21,42 ± 0,42	21,62 ± 0,32
	Neut Y	27,86 ± 0,16	27,67 ± 0,17	27,84 ± 0,19
	MNx	16,00 ± 0,29	15,58 ± 0,27	16,50 ± 0,45
	MNy	11,71 ± 0,96	12,50 ± 0,00	10,17 ± 1,33
	%MN	73,97 ± 8,33	75,18 ± 5,40	73,47 ± 8,51
	%PMN	25,63 ± 8,36	24,45 ± 5,42	25,97 ± 8,59
	Cellular HGB(g/dL)	14,84 ± 0,50	14,35 ± 0,65	14,63 ± 0,52

Supplementary Table 2. Comparative blood biochemical analysis of TAAR9-KO and WT rats. Data are Mean ± SEM, n=7 per group.

Parameters (units)	WT	TAAR9-KO^{insA}	TAAR9-KO^{delC}
Lipase (IU/L)	11.76 ± 0,92	7.89 ± 1,24	13.52 ± 3,19
Lactate dehydrogenase (U/L)	399.29 ± 46,07	255.00 ± 15,48	367.33 ± 47,10
Total protein (g/L)	63.51 ± 2,48	53.47 ± 3,21	60.27 ± 2,20
Phosphorus (mmol/L)	2.57 ± 0,13	2.21 ± 0,22	2.26 ± 0,08
Glucose (mmol/L)	7.73 ± 0,44	7.30 ± 0,42	7.72 ± 0,48
Creatinine (umol/L)	70.56 ± 17,06	44.76 ± 3,38	69.67 ± 10,73
Total bilirubin (umol/L)	1.65 ± 0,18	1.53 ± 0,24	1.33 ± 0,12
Albumin (g/L)	28.51 ± 0,87	25.51 ± 1,27	27.50 ± 0,77
Urea (mmol/L)	8.87 ± 1,27	6.00 ± 0,22	10.00 ± 1,13

Supplementary Meta-analysis 1. TAAR9 expression in organs involved in cholesterol metabolism regulation (RNAseq datasets meta-analysis).

Cholesterol concentration in plasma depends on exogenous and endogenous sources under complex regulation. Approximately half of the dietary cholesterol entering the intestines is absorbed [1]. The liver plays the main role in cholesterol metabolism. In this organ, cholesterol contributes to *de novo* synthesis and storage. High-density lipoprotein formation, cholesterol-containing chylomicron remnants and low-density lipoprotein particles uptake from plasma also occur in the liver [2]. Cholesterol up taking in hepatocytes is also insulin-dependent [3]. The inverse association between insulin and LDL cholesterol plasma levels is described both in normoglycemic and diabetic subjects [4]. The liver is also the principal site of cholesterol excretion, converting cholesterol to bile acids and removing free cholesterol as neutral sterols via biliary excretion [5]. Circulating levels of leptin showed a significant positive correlation with LDL-cholesterol and a negative correlation with HDL-cholesterol [6], so the cholesterol metabolism depends also on adipokines from adipose tissue. To estimate possible TAAR9 contribution in cholesterol metabolism regulation, its expression was evaluated in public transcriptomic data for intestine, liver, pancreatic islets, and adipose tissue.

Data collection and inclusion criteria for datasets

Publically available transcriptome RNAseq datasets were retrieved from GEO (Gene Expression Omnibus [9]) repository. First, the GEO browser available on https://www.ncbi.nlm.nih.gov/geo/browse/ was searched for the terms “intestine”, “duodenum”, “jejunum”, “ileum”, “liver”, “Langerhans islets”, “pancreatic islets”, “fat” or “adipose tissue”. Datasets were filtered by species “*Homo sapiens*” or “*Rattus norvegicus*”. Datasets inclusion criteria were as follows: at least 3 samples from healthy adult subjects or animals, the mean number of gene reads per sample at least 35 M (if SRA data available), and a file with the raw or normalized data attached.

Taar9 expression data also were searched in Expression Atlas [10] database (available at https://www.ebi.ac.uk/gxa/home), and Tabula Muris (available at https://tabula-muris.ds.czbiohub.org, [11]).

RNAseq data processing

After exclusion of all non-relevant datasets, 19 RNAseq datasets were selected for future analysis. GSE IDs and titles data for selected microarray-generated and NGS-generated datasets are listed in Supplementary Table S3. Count or normalized count matrices for these datasets were downloaded from its supplementary data on GEO Accession Display. Data were TMM normalized and CPM transformed by the Bioconductor R package edgeR [2] if necessary. TAAR9 or Taar9 expression data were extracted from TMM normalized CPM data for human or rat datasets, respectively.

Results

The following human datasets met the inclusion criteria: intestine — 1 dataset, liver — 3, Langerhans islets — 2, and adipose tissue — 6. Additionally, included rat datasets were as follows: liver — 5 and adipose tissue — 2. Only tissue samples from healthy human or non-treated animals were reviewed. No TAAR9 expression was detected in intestinal and liver samples. TAAR9 expression wasn’t detected in rat adipose tissue samples, but it was positive in 15 of 520 (3%) human adipose tissue specimens. In human pancreatic islets, TAAR9 expression was detected in 99% of specimens; only one sample in GSE165121 [10] dataset was TAAR9 expression negative.

Using the Expression Atlas thresholds [11] as a guide, the TMM normalized CPM values were below the cutoff in all adipose tissue specimens in analyzed datasets (i.e. CPM < 0.05). In all islet samples in GSE108072 and one sample in GSE165121 [10] datasets was expressed below Expression Atlas cutoff. So 28 islet specimens in GSE165121 [10] were TAAR9 expression positive at low levels according to Expression Atlas thresholds (i.e. between 0.5 to 10 CPM).

TAAR9 expression wasn’t detected in the human gastrointestinal tract, liver, pancreas, or adipose tissue in the Expression Atlas database [11]. In mice, the low expression below cutoff was detected in the liver only [11].

Using scRNA-seq data generated by the Tabula Muris we found expression of TAAR9 in mouse epithelial cells of the large intestine. Interestingly, most of the cells do not express TAAR9. Only 17 of 2019 cells (0.84%) are positive for TAAR9 expression. The median expression level 4.2 CPM in this cell group may be considered as low positive according to Expression Atlas thresholds (i.e. between 0.5 to 10 CPM).

Taking into account low TAARs expression in tissues and heterogeneous distribution of TAAR9 mRNA between the cells demonstrated in the Tabula Muris dataset, rare TAAR9-positive cases and its

controversial values in fat tissue and Langerhans islets aren't negligible for TAAR9 expression estimation.

References

1. Singh, T.; Sarmiento, L.; Luan, C.; Prasad, R.B. et al. MafA Expression Preserves Immune Homeostasis in Human and Mouse Islets. *Genes (Basel)*. **2018**, 9, 644, doi: 10.3390/genes9120644
2. Papatheodorou, I.; Moreno, P.; Manning, M.; Fuentes, A.M.-P.; George, N., Fexova, S. et al., Expression Atlas update: from tissues to single cells. *Nucleic Acids Research*. **2019**, D1, D77–D83, https://doi.org/10.1093/nar/gkz947
3. Barrett, T.; Wilhite, S.E.; Ledoux, P.; Evangelista, C.; Kim, I.F.; Tomashevsky, M.; Marshall, K.A.; Phillippy, K.H.; Sherman, P.M.; Holko, M.; Yefanov, A.; Lee, H.; Zhang, N.; Robertson, C.L.; Serova, N.; Davis, S.; Soboleva, A. NCBI GEO: archive for functional genomics data sets--update. *Nucleic Acids Res*. **2013**, 41, D991-5, doi: 10.1093/nar/gks1193.
4. Robinson, M.D.; McCarthy, D.J.; Smyth, G.K. "edgeR: a Bioconductor package for differential expression analysis of digital gene expression data." *Bioinformatics.* **2010**, 26, 139-140. doi: 10.1093/bioinformatics/btp616.
5. The Tabula Muris Consortium., Overall coordination., Schaum, N. et al. Single-cell transcriptomics of 20 mouse organs creates a Tabula Muris. *Nature*, **2018**, 562, 367–372, doi: //doi.org/10.1038/s41586-018-0590-438/s41586-018-0590-4.
6. Imes, C.C.; Austin, M.A. Low-density lipoprotein cholesterol, apolipoprotein B, and risk of coronary heart disease: from familial hyperlipidemia to genomics. *Biol Res Nurs*. **2013**, 15, 292-308. doi: 10.1177/1099800412436967.
7. Goldstein, J.L.; Brown, M.S. Regulation of the mevalonate pathway. *Nature*. **1990**, 343, 425-30. doi: 10.1038/343425a0.
8. Ramakrishnan, G.; Arjuman, A.; Suneja, S.; Das, C.; Chandra, N.C. The association between insulin and low-density lipoprotein receptors. *Diab Vasc Dis Res*. **2012**,9, 196-204. doi: 10.1177/1479164111430243.
9. Strandberg, T.E.; Tilvis, R.S.; Lindberg, O.; Valvanne, J.; Sairanen, S.; Ehnholm, C.; Tuomilehto, J. High plasma insulin is associated with lower LDL cholesterol in elderly individuals. *Atherosclerosis*. **1996**, 121, 267-273. doi: 10.1016/0021-9150(95)05733-1.
10. Turner, S.; Voogt, J.; Davidson, M.; Glass, A.; Killion, S.; Decaris, J.; Mohammed, H.; Minehira, K.; Boban, D.; Murphy, E.; Luchoomun, J.; Awada, M.; Neese, R.; Hellerstein, M. Measurement of reverse cholesterol transport pathways in humans: in vivo rates of free cholesterol efflux, esterification, and excretion. *J Am Heart Assoc*. **2012**, 1:e001826. doi: 10.1161/JAHA.112.001826.
11. Slama, F. B.; Jridi, N.; Rayana, M. C. B.; Trimeche, A.; Hsairi, M.; Belhadj, O. Plasma levels of leptin and ghrelin and their correlation with BMI, and circulating lipids and glucose in obese Tunisian women, *Asian Biomedicine*. **2015**. *9*, 161-168. doi: https://doi.org/10.5372/1905-7415.0902.382

Supplementary Table 3. RNAseq datasets included into meta-analysis

GEO ID	Authors	Title	Structure	Samples from healthy human / untreated animals, n	Species
GSE146190	Zorro M et al, 2021	RNA sequencing derived from intestinal biopsies from healthy controls and celiac disease patients	Intestine	5	Human
GSE130970	Hoang SA et al, 2019	Gene expression predicts histological severity and reveals distinct molecular profiles of nonalcoholic fatty liver disease	Liver	6	Human
GSE126848	Suppli MP et al, 2019	Hepatic transcriptome signatures in patients with varying degrees of non-alcoholic fatty liver disease compared to healthy normal-weight individuals	Liver	14	Human
GSE113024	He X et al, 2018	Next Generation Sequencing Facilitates Quantitative Analysis of conventional and ischemia-free liver transplantation Transcriptomes	Liver	12	Human
GSE124819	Chariker JH et al, 2019	Activity-induced changes in the liver transcriptome after chronic spinal cord injury	Liver	37	Rat
GSE120804	Huang X et al, 2019	Molecular Characterization of a Precision-Cut Rat Liver Slice Model for the Evaluation of Anti-Fibrotic Compounds	Liver	19	Rat
GSE123987	Vinnakota KC et al, 2019	Network Modeling of Liver Metabolism to Predict Plasma Metabolite Changes During Short-Term Fasting in the Laboratory Rat: Liver Transcriptome Changes in Study 3	Liver	16	Rat
GSE139098	Johnson KJ et al, 2020	Transcriptional Profiling of Adult Male Rat Liver and Testis following 14 Day Myclobutanil Exposure	Liver	6	Rat
GSE138454	Osuru HP et al, 2020	Impact of inflammation on hepatic subcellular energetics in anesthetized rats	Liver	8	Rat
GSE165121	Serion P et al, 2021	Transcriptional analysis of islets of Langerhans from organ donors of different ages	Langerhans islets	30	Human

GSE108072	Singh T et al, 2018	Global transcriptomic analysis of human pancreatic islets [RNAseq]	Langerhans islets	88	Human
GSE140122	Boissier R et al 2020	Molecular and cellular signature of perirenal adipose tissue	Adipose tissue	10	Human
GSE104674	Stenvers DJ et al 2019	The diurnal rhythm of adipose tissue gene expression is reduced in obese patients with type 2 diabetes	Adipose tissue	6	Human
GSE113764	U Din M, 01	Transcriptome of human white and brown adipose tissue biopsies	Adipose tissue	15	Human
GSE156044	Ha CW et al, 2020	Transcriptomics analysis of mesenteric adipose tissue from Crohn's disease patients and healthy controls [RNA-seq]	Adipose tissue	4	Human
GSE152991	Cifarelli V et al, 2020	Decreased adipose tissue oxygenation is associated with insulin resistance in people with obesity	Adipose tissue	474	Human
GSE135134	El-Sayed Moustafa JS et 10, 2020	Subcutaneous adipose gene expression from men in the METSIM cohort	Adipose tissue	4	Human
GSE165369	Kumar R et all, 2020	RNAseq of perivascular adipose tissue from mesenteric arteries from normotensive and hypertensive Dahl S rats	Adipose tissue	9	Rat
GSE161261	Wang J et all, 2020	Transcriptomic analysis of mRNA of inguinal white adipose tissues from ZSF1 lean and obese rats, ZSF1 obese rats under calorie restriction, as well as ZSF1 obese rats dosed with Cmpd1.	Adipose tissue	15	Rat
